# Application of Anodic Titanium Oxide Modified with Silver Nanoparticles as a Substrate for Surface-Enhanced Raman Spectroscopy

**DOI:** 10.3390/ma16165696

**Published:** 2023-08-19

**Authors:** Mateusz Czerwiński, Ruben del Olmo Martinez, Marta Michalska-Domańska

**Affiliations:** Institute of Optoelectronics, Military University of Technology, Kaliskiego 2, 00-908 Warsaw, Poland; ruben.martinez@wat.edu.pl

**Keywords:** titanium anodization, anodic titanium oxide (ATO), silver nanoparticles, silver electrodeposition, colloidal silver deposition, surface-enhanced Raman spectroscopy (SERS)

## Abstract

The formation of nanostructured anodic titanium oxide (ATO) layers was explored on pure titanium by conventional anodizing under two different operating conditions to form nanotube and nanopore morphologies. The ATO layers were successfully developed and showed optimal structural integrity after the annealing process conducted in the air atmosphere at 450 °C. The ATO nanopore film was thinner (1.2 +/− 0.3 μm) than the ATO nanotube layer (3.3 +/− 0.6 μm). Differences in internal pore diameter were also noticeable, i.e., 88 +/− 9 nm and 64 +/− 7 nm for ATO nanopore and nanotube morphology, respectively. The silver deposition on ATO was successfully carried out on both ATO morphologies by silver electrodeposition and Ag colloid deposition. The most homogeneous silver deposit was prepared by Ag electrodeposition on the ATO nanopores. Therefore, these samples were selected as potential surface-enhanced Raman spectroscopy (SERS) substrate, and evaluation using pyridine (aq.) as a testing analyte was conducted. The results revealed that the most intense SERS signal was registered for nanopore ATO/Ag substrate obtained by electrodeposition of silver on ATO by 2.5 min at 1 V from 0.05M AgNO_3_ (aq.) (analytical enhancement factor, AEF ~5.3 × 10^4^) and 0.025 M AgNO_3_ (aq.) (AEF ~2.7 × 10^2^). The current findings reveal a low-complexity and inexpensive synthesis of efficient SERS substrates, which allows modification of the substrate morphology by selecting the parameters of the synthesis process.

## 1. Introduction

Due to the interesting properties of anodic titanium oxide (ATO), such as corrosion resistance, high porosity, developed surface area, semiconductive properties, high stability, and mechanical resistance [1], ATO is a unique nanomaterial in material engineering, medicine, and photocatalysis fields [2,3,4,5,6,7].

Anodization is a cost-effective and efficient method to form nanostructures on titanium and its alloys. The main advantage is the control over the resulting morphology (ATO thickness as well as the inner and outer diameter of nanotubes/nanopores) by modifying the anodization parameters and the electrolyte composition [8,9]. After anodizing, the electrolyte properties, such as the concentration of ions, pH value, and electrolyte conductivity change. This is well-known as electrolyte aging strongly affects ATO morphology [10]. The most common electrolytes used for Ti anodization are composed of an inorganic salt of fluoride, an organic solvent (like ethylene glycol), and water [11,12]. Nowadays, also, new types of electrolytes solvents for Ti anodization are developed, for example, ethanol and glycerol beside ethylene glycol [13,14]. Another interesting feature of Ti anodization is the possibility of obtaining different ATO morphologies by determining/changing the concentration of fluoride ions in the electrolyte. Generally, higher fluoride ions concentration leads to the formation of nanotube oxide morphology (>0.2 M), and lower F^−^ content in electrolyte results in ATO nanopore morphology (0.05 M–0.15 M). Nevertheless, the optimal proportion between mentioned anodization parameters and electrolyte composition should be determined for the specific conditions and devices setup to provide replicable ATO morphology. 

Although the abovementioned factors are critical to forming self-ordered ATO morphology, the conduction of the annealing process is required to obtain crystal structure from primary amorphous ATO. Generally, anodic titanium oxide appears in three polymorphic forms: anatase, rutile, and brookite [15]. According to different studies, anatase demonstrates the best photocatalytic properties (delayed carrier charges recombination) and has the lowest surface enthalpy and surface energy among ATO polymorphs [16,17]. However, it should be remembered that depending on the type of chemical reaction carried out, other phases (rutile) or combinations of phases (mixture of anatase and rutile) of crystalline ATO may be preferred as the best catalyst [18]. In general, at 400 °C, amorphous ATO, at first, transforms to metastable anatase [19]. At temperatures between 400 °C and 700 °C, rutile simultaneously starts to form, and a mixture of anatase and rutile is present in resulted material [19]. Next, by raising the temperature up to 1200 °C, rutile obtains the predominant crystal phase. Brookite occurs in nature, but due to its low stability and conversion to rutile, it is detectable only in traces or not at all. Depending on the annealing regime (i.e., annealing temperature, time, atmosphere of annealing, pressure, and elements doping) in many scientific experiments, various ratios of anatase–rutile mixture are obtained [20]. On the other hand, temperature gradient and cooling rate are important in terms of the mechanical stability of resulted ATO surface [21].

Among the many possible applications, the use of ATO as a substrate in surface-enhanced Raman spectroscopy has attracted a great deal of attention over the last several years [22]. In order to obtain an enhancement of the signal by inelastic scattering of IR radiation, it is required to use a metal with the negative value of the real part of electrical permeability, i.e., silver, gold, or copper [23]. Plasmons are created on the metal/dielectric interface when the light of a specific wavelength interacts with the plasmonic metal of the nanometer order of magnitude. The Raman active molecule, adsorbed on the surface covered with rough plasmonic metal, has enhanced response in Raman signal by chemical mechanism (contribute 10^2^ enhancement) and electromagnetic mechanism (up to 10^12^ enhancement) [24]. As-prepared, non-modified anodic titanium oxide cannot be applied as a SERS substrate due to the lack of plasmonic properties [25]; therefore, deposition of plasmonic nanoparticles on the ATO surface is required. Most research is aimed at the deposition of silver and gold at various substrates. Generally, due to the developed ATO surface area, the possible surface area for Ag deposition increases. Furthermore, specific ATO surface geometry and interaction between ATO surface and Ag nanoparticles could result in a more prominent SERS signal for ATO/Ag, compared to commercially available SERS substrates (e.g., ITO/Ag) [26]. Composites such as ATO/Ag exhibit localized surface plasmon resonance at visible light (VIS) and near-infrared range(NIR). For example, rhodium and ruthenium nanoparticles are the candidates for possible enhancement of signal in the ultraviolet (UV) range [27]. In comparison to the UV range, SERS measurements using VIS and NIR lasers have two major advantages: fluorescence signal ratio is much lower and VIS lasers are much more profitable. The condition for the occurrence of surface plasmon resonance is the difference in the electrical permeability of the two media. In most research, a light wave in the VIS range passes from a gaseous medium with positive electrical permeability to a solid medium (e.g., Ag nanoparticles) with negative electrical permeability in the VIS range [28]. For most applications, the 532 nm or 785 nm lasers paired with either silver or gold nanoparticles SERS substrate is sufficient to prompt surface plasmon resonance in the VIS region [29]. Another important SERS substrate parameter is the repeatability of the surface topography. Although the great emphasis on the control of the topography that mimics highly roughen surfaces was performed in the last decade, poor measurement repeatability and site-dependent enhancement factor are still problems that need to be addressed in novel research [27]. Research related to the control of ATO/Ag substrate morphology using low-cost surface modification techniques can contribute to improvements in detection parameters for SERS on ATO substrates as well as the lowering of fabrication and distribution costs of single SERS substrates. Generally, the SERS substrates available commercially have uniform surface coverage and give significant SERS signal enhancement. On the other hand, the high cost of single substrate and expensive processing devices used in its fabrication processes (usually physical vapor deposition (PVD) or lithographic methods) are major problems that need to be solved to make SERS spectroscopy more accessible to a wider group of analysts [30]. Those conditions can be achieved by Ti anodizing, and that is why we present this approach.

For this reason, considering the abovementioned advantages of Ti anodizing, in this research, two different ATO morphologies are addressed: nanopore and nanotube. Then, two cost-effective and barely addressed silver deposition methods will be performed on ATO: electrodeposition and colloidal deposition. In this research, we will focus on silver nanoparticles because of their attractive costs, accessibility, and good results observed in other SERS studies [31,32]. 

Usually, in scientific articles, a test molecule is chosen to assess the parameters of the substrate (i.e., enhancement factor), or specific organic compounds are analyzed on a well-studied substrate. A prerequisite to occur the inelastic scattering is a change in the polarizability of the tested molecule. Therefore, the most studied groups of molecules are those containing an aromatic ring, amino or thiol groups (i.e., 4-merkaptobenzoic acid [33], cysteine [34]), porphyrins [35], dyes (i.e., Rhodamine 6G [36]), and peptides (i.e., Trp-Trp, Gly-Gly-Gly [37]). Based on the large number of studies present in the literature, as well as the specific molecule structure (the presence of an aromatic ring with a nitrogen atom), pyridine was chosen as the test molecule for SERS measurements.

Generally, in the case of anodic titanium oxide, morphology, type, shape, and distribution of plasmonic nanoparticles on its surface are the most important components that could have an influence on the obtainable enhancement factor in SERS. Deposition of close-packed Ag nanoparticles with narrow edges on the ATO matrix could be highly preferable. Furthermore, presented electrodeposition techniques allow control over the distribution of silver nanoparticles on the ATO surface [38,39]. Considering the gap in knowledge regarding the insufficient amount of information about the effect of deposition parameters on the ATO/Ag interface, in the presented study, we evaluate the effect of Ti anodization parameters and Ag deposition parameters on the morphology and uniformity of ATO/Ag composites. Different silver deposition parameters were studied, and trials using ATO/Ag composites as potential SERS substrates were conducted. 

In this research, a comparison of ATO/Ag systems with different Ag morphology, obtained by electrochemical deposition and deposition from colloid solution, was presented. The novelty of this research is the preparation of an active and cost-effective SERS substrate with a relatively high enhancement factor. Moreover, the low complexity of the presented ATO/Ag systems preparation (electrodeposition and deposition from colloid solution) compared to other SERS substrate preparation techniques (expensive and complicated PVD or lithography techniques) is the advantage of the presented SERS composite substrate.

## 2. Materials and Methods

### 2.1. Synthesis of Nanostructured ATO

Titanium sheets (2 × 1.5 × 0.025 cm^3^) of 99.5% purity (Alfa Aesar, Ward Hill, MA, USA) were used as the starting material to obtain anodic titanium oxide. Ti samples act as the anode, and a platinum grid was used as the cathode during the anodizing process. Prior to anodizing, titanium samples were cleaned with a 1:1 mixture of acetone and 2-propanol in an ultrasonic cleaner (Bandelin Sonorex DT 52, Berlin, Germany). The working area of the titanium electrode (3 cm^2^) was reduced by using electrochemical-resistant tape (3 M). The experimental system used during the anodization process consisted of the following components: glass cell, power supply (NDN DF1760SL10A, Warsaw, Poland), multimeter (Rigol DM3058E, Warsaw, Poland), thermostat (Huber MPC K6, Offenburg, Germany), magnetic stirrer (Heidolph, Schwabach, Germany). For each anodization process, a current density–time curve was registered with a computer. Ethylene glycol (Chempur, Piekary Śląskie, Poland), ammonium fluoride (Sigma Aldrich, Burlington, MA, USA), and demineralized water (Hydrolab, Straszyn, Poland) were used to prepare the electrolyte solutions. Based on the experiments and literature analysis, an ethylene glycol-based electrolyte with 0.05 M NH_4_F and 2 vol.% H_2_O content was used to obtain ATO with nanopore morphology. Anodization was carried out for 30 min at a voltage of 40 V and at a temperature of 30 °C. For ATO with nanotube morphology, an ethylene glycol-based electrolyte with 0.45 M NH_4_F and 3 vol.% H_2_O was applied for 1 h using 40 V voltage and 20 °C. During anodization, the electrolyte solutions were stirred at 500 rpm. After the anodization process, the insulating tape was removed, and the titanium sheets were thoroughly washed in demineralized water.

In order to obtain crystalline ATO, the samples were annealed at 450 °C for 2 h with a temperature rate of 5 °C/min. After annealing, samples were cooled down to room temperature inside the furnace to avoid surface cracking phenomena due to the possible thermal shock.

### 2.2. Silver Deposition on ATO Samples

The silver deposition processes were conducted on nanopores and nanotubes ATO specimens by two methods. Silver electrodeposition was carried out in a two-electrode system at room temperature. The anodized titanium sheet served as the cathode, while the platinum grid served as the anode. The electrodes were connected to an NDN DF1760SL10A power supply. Voltage and time were constant for each sample and were determined in the preliminary study to 1 V and 2.5 min, respectively. Three aqueous solutions of AgNO_3_ were prepared (0.1 M, 0.05 M, and 0.025 M) for the electrodeposition process. After silver electrodeposition, the ATO/Ag samples were gently rinsed in water for 1 min. 

**Figure 1:**
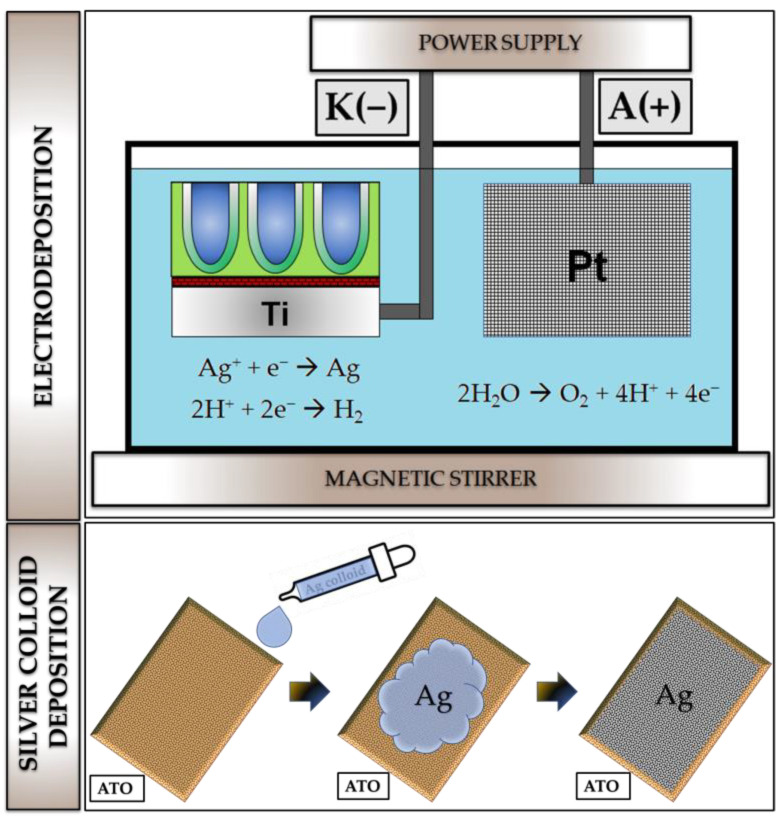
Graphical presentation of methods that were used for deposition of Ag on ATO surface.

In the available literature, reports focused on silver colloid deposition on non-ATO substrates are present [40,41]. There are many possible routes for the synthesis of silver colloids. The most commonly used is the reaction with an organic reductant (to allow the reduction of silver (I) to silver (0)) and a stabilizer (to prevent agglomeration of the silver nanoparticles). Generally, silver in ionic form in solution is reduced to metallic silver to form a colloid solution. Three different silver colloid solutions were prepared to deposit the silver colloid on the ATO surface, i.e., 0.5% trisodium citrate + 0.2 M AgNO_3_ (1), 5% trisodium citrate + 0.2 M AgNO_3_ (2), and 0.5% trisodium citrate + 0.2 M [Ag(NH_3_)_2_]^+^ (3). A calculated amount of water was added to a conical flask and brought to a boil. Then the abovementioned concentration of silver nitrate and trisodium citrate was added to the flask. This mixture was boiled for 40 min, and then the contents were centrifuged for 15 min at 4000 rpm. In reaction to silver colloids fabrication, trisodium citrate acts as a reducing agent and a stabilizer of silver particles. The prepared silver colloids were shaken (IKA, Vortex 1, Warsaw, Poland) and then used for deposition on the ATO surface. On each ATO sample, 20 μL of the colloid solution was deposited with a micro-pipette and left to the air solvent evaporation. Figure 1 shows the graphical scheme of the used deposition routes for silver deposition on the ATO surface.

### 2.3. Characterization of ATO and ATO/Ag Substrates

Scanning Electron Microscope (SEM, FEI Quanta 3D FEG, Thermo Fisher Scientific, Waltham, MA, USA) was used to analyze the surface morphology of ATO before and after silver deposition. 

Raman spectroscopy was applied to confirm the crystal structure of ATO nanotubes and nanopores. ImageJ program [42] served to calculate ATO inner diameter, surface coverage of samples ATO/Ag, and the size of Ag nanoparticles.

The SERS measurements were carried out using i-Raman Plus (Metrohm, Herisau, Switzerland) with a 785 nm laser in the range of wavelength of 800–1500 cm^−1^. The referential SERS measurements in the same conditions were performed on the commercially available SERS substrate manufactured by SERSitive (S-silver SERS substrate, Warsaw, Poland). Raman measurements were performed on the Ag/ATO systems using 5 vol.% pyridine (aq.), while SERS measurements were carried out using 0.1 vol.% pyridine (aq.). An analytical signal enhancement factor (AEF) was used to evaluate the Raman signal intensity enhancement. The AEF indicates how much more signal can be expected from SERS compared to classical Raman under the same measurement conditions. This specific type of enhancement factor is useful to evaluate how much the signal has been enhanced by the SERS technique in relation to classical Raman spectroscopy. In this research, the AEF was used to compare, with a certain margin of error, different SERS substrates.

On each of the prepared ATO/Ag samples, 20 µL of the tested solution was deposited. Next, H_2_O was evaporated, and SERS measurements were conducted in three different regions of a particular sample. Analytical enhancement factor (AEF) was estimated with reference to breathing pyridine mode at 1030 cm^−1^ using the following Equation (1):

AEF = (ISERS/C_SERS_)/(I_RS_/C_RS_) (1)

where ISERS and I_RS_ are intensities of signal in SERS, and classical Raman, C_SERS_ and C_RS_, are pyridine concentrations in SERS and Raman, respectively. This form of enhancement factor can be used when measurements are conducted under the same parameters, such as laser wavelength, laser power, and microscope objective [43,44]. 

## 3. Results and Discussion

### 3.1. Anodic Titanium Oxide Formation

**Figure 2:**
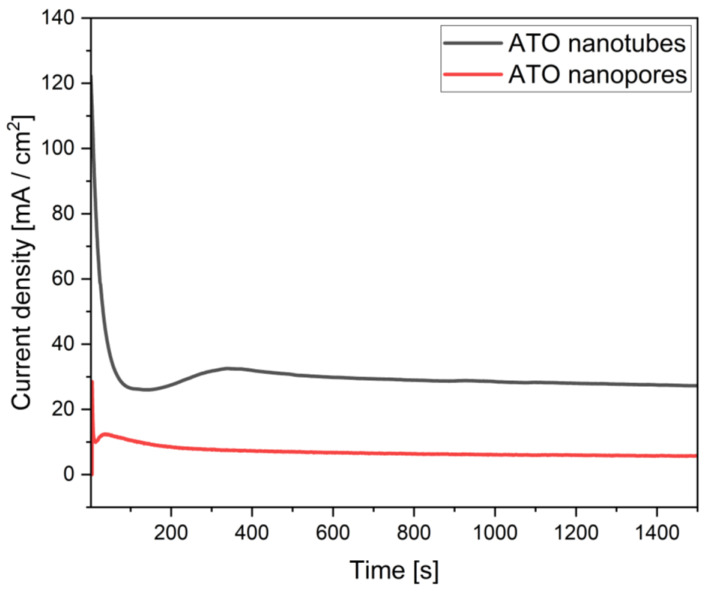
Current density–time curves for anodizing Ti at 40 V to form self-ordered nanopores and nanotubes ATO layers. Plotted data correspond to one representative specimen of 6 replicas. Note that the plotted treatment time corresponds to the first 1500 s of the anodization for ease of comparison.

Figure 2 shows the current density–time curves for Ti anodized at 40 V to form self-ordered nanotube and nanopore ATO layers. Due to the different electrolytes and experimental conditions, the current density–time signal intensity and shape of both types of recorded curves show several differences. In detail, the formation of such nanopore and nanotube ATO layers in F-containing electrolytes occurs in three expected stages, as numerous studies reported and explained by the plastic flow model and field-assisted dissolution models [45,46,47,48]. 

For ATO nanopore morphology, in stage I (0–20 s) (Figure 2), a noticeable decrease in the recorded current density (from ~29 mA/cm^2^ to ~10 mA/cm^2^) was observed. By contrast, in nanotube ATO layers, in stage I (0–20 s) (Figure 2), the shape of the curve and current density value (~124 to 27 mA/cm^2^) were consistently different compared to the nanopore ATO layer (Figure 2). In both cases, the observed current density decrease is usually associated with the formation of a dense and highly resistive barrier oxide layer [49]. Considering that the anodizing of both samples was performed at 40 V, the higher current density recorded in the nanotube ATO specimen formation is mainly related to the high (i) NH_4_F concentration in the electrolyte and (ii) the electrolyte temperature [50]. The concentration of free NH_4_^+^ and F^−^ ions in the electrolyte for the production of nanotube ATO (i.e., 0.45 M) was superior to the solution for the fabrication of nanopore ATO layers (0.05 M), thus resulting in higher ionic conductivity (2263 μS/cm and 345 μS/cm for 0.05 M NH_4_F-2 vol.% H_2_O (nanopore ATO formation) and 0.45 M NH_4_F-3 vol.% H_2_O (nanotube ATO formation), respectively. Another factor to consider is the electrolyte temperature. According to previous studies [51,52,53], a higher electrolyte temperature favors the dissociation of the NH_4_F and increases the ionic mobility of the electrolyte solution, thus decreasing the viscosity of the electrolyte.

In stage II (approximately from 20 to 400 s), in the case of electrolyte for both ATO morphologies formation, there is a slight increase in the recorded value of the current density. This phenomenon is due to the formation of nanopores in a dense barrier oxide layer by the reaction of the fluoride anions with the formed oxide layer [54]. Although this is also related to the formation of cracks and nanopores in the oxide barrier layer, several studies reported that the electromagnetic field and the mobility of the ions in the anode surroundings are proportional to the F^−^ concentration in the electrolyte, thus leading to the characteristic nanotube ATO morphology [55]. 

In stage III (from 400 s), current density–time curves show stable current values of ~12 mA/cm^2^ and ~30 mA/cm^2^ for nanopore and nanotube ATO morphology, respectively. This is due to the establishment of oxide growth–dissolution equilibrium owing to the presence of fluoride anions in both types of electrolytes. Note that Figure 2 shows the treatment time up to the first 1500 s of the anodization for ease of comparison. However, a longer treatment time (i.e., 3600 s) is needed to ensure the formation of nanotubes on the Ti substrate [56]. Therefore, the anodic oxide layer thickness, typically related to the charge passed through the forming oxide formation, was higher for nanotube (3.3 ± 0.6 µm) than nanopore ATO layers (1.2 ± 0.3 µm). 

**Figure 3:**
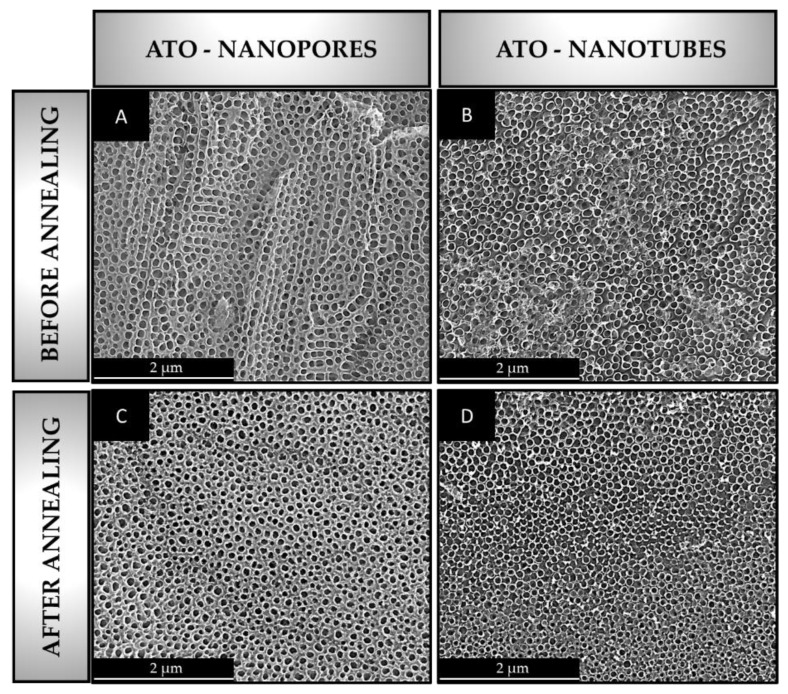
SEM images of nanopore and nanotube ATO morphologies before and after annealing process at 450 °C. ATO nanopores before annealing (**A**), ATO nanotubes before annealing (**B**), ATO nanopores after annealing (**C**), and ATO nanotubes after annealing (**D**).

The morphology of the resultant nanopore and nanotube ATO layers before and after annealing is shown in Figure 3.

The nanopore ATO samples show a uniform surface appearance and a high self-ordering degree (Figure 3A). Moreover, the nanotube ATO specimens also showed a high degree of self-ordering, and several heterogeneous regions were founded over the surface. This may be due to the high concentration of NH_4_F in the electrolyte [57], as a high F^−^ ions concentration (over 0.3 M) is known to promote the dissolution of the oxide formation process of the anodic film, thus leading to the slightly collapsed nanotube region on the ATO surface. 

Since the experimental conditions were carefully optimized in previous studies [58], homogeneous internal diameters were formed in both cases. In detail, the inner diameter of nanopore and nanotube ATO oxide layers were 88 ± 9 and 64 ± 7 nm, respectively. 

In addition, note that the annealing conditions used in this study did not cause cracks or surface damage to the resultant ATO layers (Figure 3C, D). This may be due to the low annealing temperature (450 °C) [59] since annealing at temperatures above 600 °C may lead to the partial cracking and collapse of the ATO nanotube walls [60]. Another factor to consider is the heating rate (5 °C/min) and cooling regime (non-air thermal shock), as non-controlled heating–cooling usually causes a pores shrinkage, thereby leading to pore damage and detached ATO layer from the Ti substrate [61].

**Figure 4:**
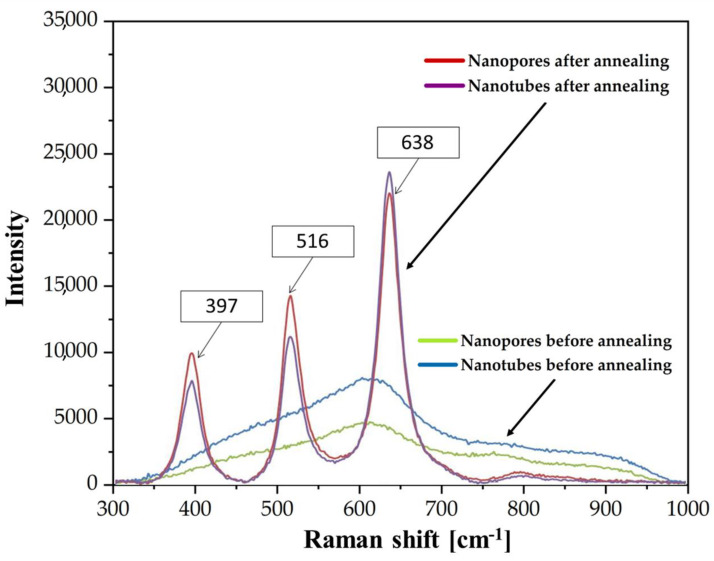
Raman spectra of ATO nanotubes and nanopores before and after annealing process conducted at 450 °C.

To confirm the formation of crystalline structures in both studied morphology of ATO samples, Raman spectroscopy was used before and after the annealing process (Figure 4). For easier identification, Table 1 provides information on the active modes of the crystallographic structures of titanium oxide. Namely, through the D$\frac{19\;}{4h}$ space group correlation method for anatase and D$\frac{14\;}{4h}$ for rutile, under the assumption of site symmetries, there are six and four active mods for anatase and rutile, respectively [62].

As can be seen in Figure 4, and as expected, non-annealed specimens are amorphous. After annealing, both studied morphologies of ATO specimens showed the characteristic peaks of anatase at 397 cm^−1^, 516 cm^−1^, and 638 cm^−1^. The characteristic Raman rutile phase peaks at 446 cm^−1^ and 610 cm^−1^ were not detected. This is in line with previous studies where anatase formation was reported at 450 °C [64]. Nevertheless, the absence of rutile may be related to the annealing time, as other studies also detected rutile at this temperature at a longer annealing time [65]. It should be noted that second-order mods and two-photon scattering reflexes can also appear in the Raman spectrum. Nonetheless, these signals were not detected in the present study. This is also beneficial for silver deposition since it has been previously reported that rutile structure may retard the formation of silver nanoparticles [66,67].

### 3.2. Silver Deposition on Anodic Titanium Oxide Layers

**Figure 5:**
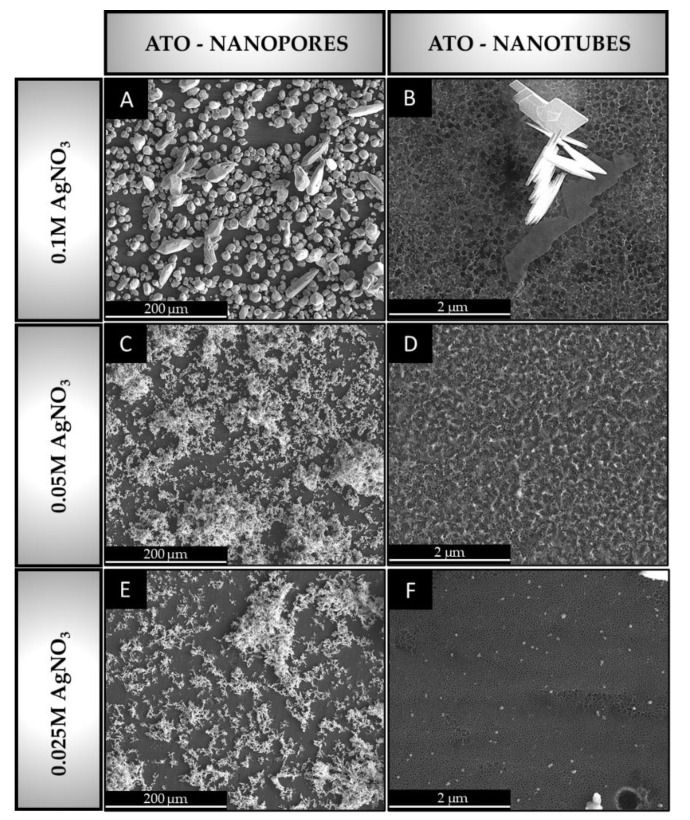
SEM images of nanopore and nanotube ATO morphologies after silver electrodeposition from electrolyte containing AgNO_3_ aqueous solution at different concentrations. ATO nanopore with silver nanoparticles deposited from electrolyte with 0.1 M AgNO_3_ (**A**), 0.05 M AgNO_3_ (**C**), 0.025 M AgNO_3_ (**E**), ATO nanotube with silver nanoparticles deposited from electrolyte with 0.1 M AgNO_3_ (**B**), 0.05 M AgNO_3_ (**D**), 0.025 M AgNO_3_ (**F**).

Figure 5 shows SEM images of nanopore and nanotube ATO samples after the cathodic silver deposition on their surface. Table 2 summarizes the morphological features of these studied ATO structures. Note that the different magnifications of SEM are due to the noticeable difference in the amount of silver deposited on both morphologies at the studied AgNO_3_ concentrations. 

For nanopore ATO morphology, the silver distribution and crystal shapes were unlike the function of the AgNO_3_ concentration in the electrolyte. The ATO surface covered by the silver electrodeposited from the electrolyte with 0.1 M AgNO_3_ results in an ATO total surface silver coverage of 65.9 ± 0.3%. However, this electrodeposition condition shows well-defined and dispersed clusters with noticeable differences in shapes and sizes. 

For silver nanoparticles formed from electrolytes with lower AgNO_3_ concentrations (0.05 M and 0.025 M), smooth ellipsoidal-shaped nanoparticles formed similarly larger clusters. In addition, surface silver distribution was comparable to that obtained from electrolytes with 0.1 M AgNO_3_ in both cases, i.e., 62.6 ± 1.1% for electrolytes with 0.05 M AgNO_3_ and 57.1 ± 2.1% for electrolytes with 0.025 M AgNO_3_. 

Nevertheless, to the authors’ best knowledge, the available literature concerning the study of the silver electrodeposition on ATO layers is quite scarce. Notwithstanding, these differences in size and distribution of nanosized deposited particles found for samples made in electrolytes with different AgNO_3_ concentrations are in line with the results found in the literature for TiN and pure silver substrates [68]. These studies reported heterogeneous silver deposition regardless of the concentration of silver used (0.01–1.07 M), which is in line with the reported findings in this study. 

Note that these procedures were performed in potassium succinate and tartaric acid for TiN and pure silver substrates, respectively. For both substrates studied, it was reported that the presence of a complexing agent in the solution used to deposit Ag favors the formation of smaller grains and silver deposits that are larger-sized in length (4–5 μm) at a suitable high Ag^+^ concentration (0.22 M) [68]. Therefore, the distinct distribution and size of silver clusters on the studied ATO specimens may be associated with the higher concentration of AgNO_3_ in the electrolyte. Note that in the present study, the absence of a reducing agent in the electrolyte was chosen to promote the smaller silver clusters to promote the maximum SERS performance. These things considered, the results show that higher AgNO_3_ concentration in an electrolyte to silver electrodeposition promoted less covered surfaces.

Regarding the silver distribution, a representative study by M. Nycz et al. [66] described the formation of silver nanoparticles deposited on anodized Ti substrate by three methods: cyclic voltammetry, chronoamperometry, and sputter deposition routes. The authors reported variable silver nanoparticle diameters (5–70 nm) as a function of the deposition method and the operating conditions as the AgNO_3_ concentration was constant (0.001 M). Although surface coverage data was not reported in this work, based on SEM examinations, the authors confirmed a homogeneous silver distribution for samples made at 25 cycles of cyclic voltammetry. Silver was embedded mostly on nanotube rings and inner ATO walls. This may be associated with the conditions used in our research since non-independent particles were detected for 0.05 M and 0.025 M of Ag-deposited samples (Figure 5C,E). Deposited nanoparticles tend to form clusters placed on top of ATO nanotubes.

In our research for ATO nanotube morphology (Figure 5B, D, F), the amount of deposited silver was minimal for all studied concentrations of AgNO_3_ in the electrolyte. More specifically, surface coverage was 12.7 ± 0.3%, 8.2 ± 1.7%, and 10.5 ± 0.9% for electrolytes in 0.1 M, 0.05 M, and 0.025 M AgNO_3_ solutions, respectively. In all samples, different sizes of single crystals were detected on the surface, independently of the used AgNO_3_ concentration. Namely, the average size of nanoparticles was equal to 2210 nm, 68 nm, and 71 nm for electrolytes with 0.1 M, 0.05 M, and 0.025 M of AgNO_3_, respectively. In addition, the comparable size of the crystals obtained from electrolytes with 0.05 M and 0.025 M AgNO_3_ highlights the lack of reproducibility of the silver electrodeposition method for this type of ATO morphology. The difference in the total Ag surface area of the deposited silver may be related to the superior layer thickness (3.3 ± 0.6 µm) of the ATO nanotube layer compared to the nanopore ATO layer (1.2 ± 0.3 µm). This is in line with the literature and the process itself, as thicker oxide layers hamper the current flow throughout the Ti substrate, thus decreasing the amount of electrodeposited silver [69].

**Figure 6:**
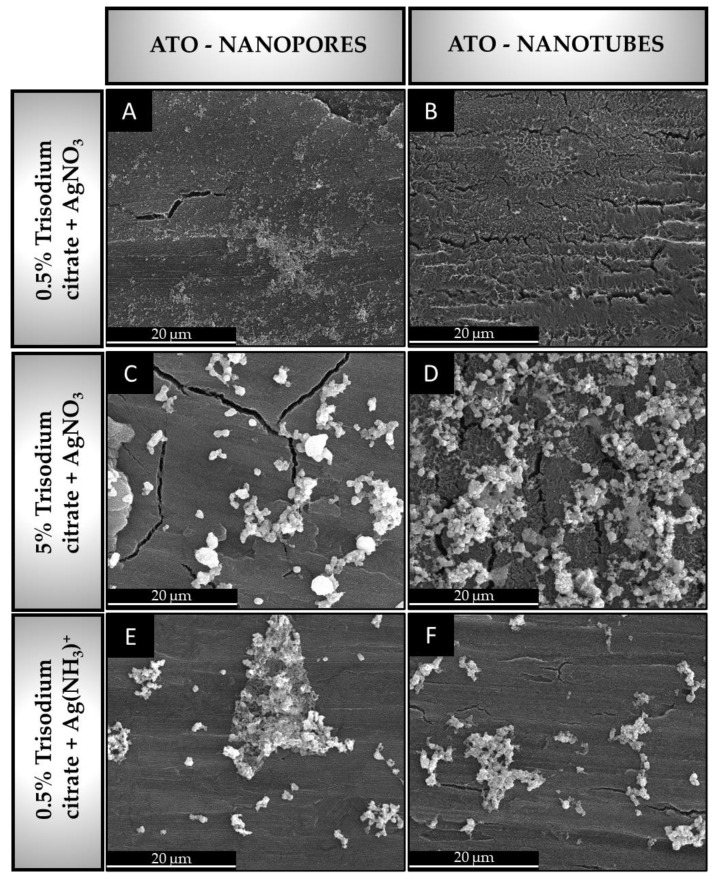
SEM images of nanopore and nanotube ATO morphologies after silver colloids deposition from solutions with different content of AgNO_3_. ATO nanopores with silver nanoparticles deposited from 0.5 wt.% trisodium citrate + AgNO_3_ (**A**), 5 wt.% trisodium citrate + AgNO_3_ (**C**), 0.5 wt.% trisodium citrate + Ag[(NH_3_)_2_]^+^ (**E**). ATO nanotubes with silver nanoparticles deposited from 0.5 wt.% trisodium citrate + AgNO_3_ (**B**), 5 wt.% trisodium citrate + AgNO_3_ (**D**), 0.5 wt.% trisodium citrate + Ag[(NH_3_)_2_]^+^ (**F**).

The SEM images of nanopore and nanotube ATO structures after silver colloid deposition are shown in Figure 6. The morphological features of these studied ATO structures are collected in Table 3.

In all samples, different percentages of ATO surface coverage were obtained as a function of: (i) the inorganic Ag salt used (AgNO_3_ or [Ag(NH_3_)_2_]^+^), (ii) the amount of organic reductant (0.5 wt.% or 5 wt.% trisodium citrate) and (iii) the type of ATO morphology. 

For both morphologies and silver sources (AgNO_3_ and [Ag(NH_3_)_2_]^+^), smaller and less distributed nanoparticles were obtained from the colloid solution used with 0.5 wt.% trisodium citrate than from solution with higher reductor concentration (5 wt.%). In the case of the AgNO_3_ salt used in the solution to colloid deposition, for ATO nanopore morphology, the surface covering and Ag cluster sizes were similar for colloids fabricated from a solution of 0.5 wt.% (Figure 6A) and 5 wt.% trisodium citrate concentrations (Figure 6C). Regarding the silver colloids deposition on ATO nanotubes, the Ag clusters with similar morphological features to that obtained on ATO nanopores were also detected for both concentrations of trisodium citrate (Figure 6B, D). This is in line with the study of K. Syrek et al. [70], where the authors reported the silver nanoparticles deposition on ATO nanopore substrate annealed at 500 °C. Silver nanoparticles of uniform size (25 ± 3 nm) were deposited using an equimolar mixture of AgNO_3_ and sodium citrate (1 mM) in ultrasounds. In addition, Ag nanoparticles were susceptible to forming larger agglomerates when the 5 wt.% trisodium citrate as a reducing agent and stabilizer was used. This may be due to insufficient stabilization of the colloid nanoparticles by the trisodium citrate at low concentrations [71]. The difference in distribution and size of the silver clusters in our research may be due not only to the higher concentration of trisodium citrate but also to the larger oxide surface area of interaction with the colloidal solution than for ATO nanopores. As can be seen in Figure 6D, silver is also deposited between the nanotubes, which could justify, to some extent, the higher deposition of silver on ATO nanotubes and, therefore, a possible synergistic effect between the higher concentration of trisodium citrate and the larger surface area of interaction between ATO and silver nanoparticles. 

These things considered, recent studies have demonstrated the formation of larger agglomerates of silver nanoparticles with several chemical deposition cycles [72]. A variable Ag agglomerate size was also reported, depending on the substrate type/morphology, by using a constant composition of the solution to silver deposition, namely AgNO_3_ (1 × 10^−3^ M) and 1 wt.% trisodium citrate. Successive repetition of the sample immersion in the colloidal solution procedure was necessary to obtain a more uniform morphology of the silver deposit. For substrates such as SiO_2_ and polyethylene terephthalate (PET) substrates, the average cluster sizes were 240 nm and 400 nm, respectively [72]. Nevertheless, a non-homogeneous morphology with high-size dispersion of silver nanoparticles was observed on both types of substrates. In the present study, the average cluster size was superior to those from the study described. Therefore, to achieve a minimal cluster size, a one-single sample immersion step was performed in the present study, thus achieving comparable results to the abovementioned substrates (Ag/SiO_2_ and Ag/PET). 

Since the deposited silver surface area was higher using a higher concentration of trisodium citrate in solution to silver deposited (5 wt.%), the authors further studied the effect of [Ag(NH_3_)_2_]^+^ as a different source of silver. The results show a different morphological feature in terms of average silver cluster sizes and surface distribution over both studied ATO morphologies (Table 3). The standard reduction potential is lower for [Ag(NH3)_2_]^+^/Ag (+0.373 V) than for Ag+/Ag (+0.7996 V) [73], so the reduction of silver from the ammonia complex is expected to be more stable than the reduction from AgNO_3_. The reaction of the silver complex with the aqueous ammonia solution is given in Equation (2).

Ag^+^ + 2NH_3_ ∙ * H_2_O → [Ag(NH_3_)_2_]^+^ + 2H_2_O (2)

Therefore, the larger size of the Ag nanoparticles produced from the aqueous solution containing [Ag(NH_3_)_2_]^+^ may be due to the larger amount of silver nuclei formed at the beginning of the deposition process. In addition, the wide range of observable diameters may be due to the combined effect of a large number of crystal nuclei in the initial phase of the deposition process and the excessive aggregation of the nanoparticles.

### 3.3. SERS Measurements for ATO/Ag Obtained Electrochemically

Due to the higher surface homogeneity and a high degree of surface coverage by Ag nanoclusters, electrochemically prepared samples on ATO substrate with nanotube morphology were selected for further studies. SERS measurements were performed on representative samples consisting of ATO nanopore morphologies covered electrochemically by Ag nanoparticles in AgNO_3_ solutions of 0.025 M, 0.05 M, and 0.1 M at 1 V. The referential SERS measurements in the same conditions were performed on the commercially available SERS substrate manufactured by SERSitive (S-silver SERS substrate). The Raman spectrum of pyridine typically exhibits several intense peaks in the range of 1000–1600 cm^−1^, which correspond to the vibrational modes of the C-C, C-H, and C-N bonds in the aromatic ring [74]. The most prominent peaks are usually located at around 1000–1050 cm^−1^ and correspond to the C-H breathing mode [75]. 

**Figure 7:**
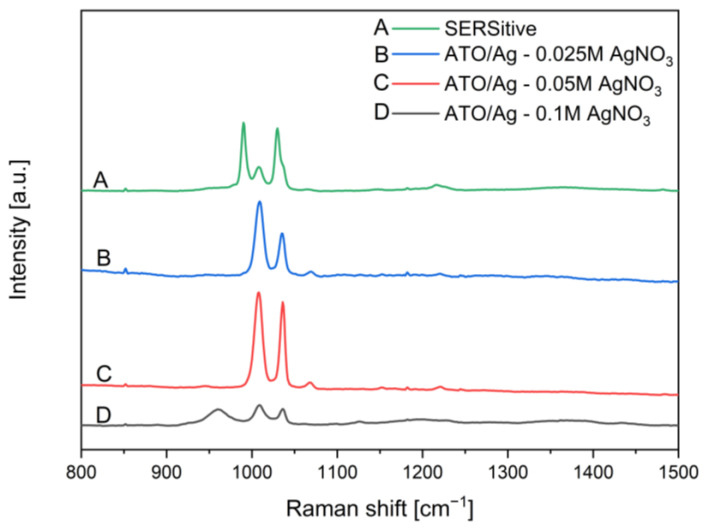
SERS spectra of pyridine registered on (A) commercially available SERS substrate—SERSitive—act as reference (B) ATO/Ag SERS substrate covered by Ag in 0.025 M AgNO_3_ solution (C) ATO/Ag SERS substrate covered by Ag in 0.05 M AgNO_3_ solution (D) ATO/Ag SERS substrate covered by Ag in 0.1 M AgNO_3_ solution.

Figure 7 shows SERS spectra of pyridine collected on ATO nanopores/Ag and SERSitive samples after background correction and normalization. Intense peaks from pyridine were observed for each tested SERS substrate. The placement of pyridine peaks differs depending on the bonds that are created between Ag nanoparticles and adsorbed pyridine molecules. Importantly, the polarizability of pyridine is changed due to adsorption. Raman breathing modes of pyridine in Raman spectroscopy are placed at 978 cm^−1^ and 1022 cm^−1^. Due to electron transfer between HOMO (Highest Occupied Molecular Orbital) of pyridine with LUMO (Lowest Unoccupied Molecular Orbital) of Ag nanoparticles, breathing modes of pyridine are slightly shifted: (A) 990 cm^−1^, 1006 cm^−1^, 1030 cm^−1^; (B) 1006 cm^−1^, 1036 cm^−1^; (C) 1006 cm^−1^, 1036 cm^−1^; (D) 960 cm^−1^, 1010 cm^−1^, 1036 cm^−1^. For two tested SERS substrates, SERSitive (A) and ATO/Ag—0.1 M AgNO_3_ (D), additional peaks appear on spectra, which may correspond to the specific geometry of the sample or orientation of adsorbed pyridine molecules with respect to the direction of the laser beam.

Overall, it is well known that the shape of silver nanoparticles affects the registered SERS signal. For instance, Vidhu S. Tiwari et al. [76] evaluated the effect of silver nanoparticles size with constant silver amount deposited on enhancement factor in SERS. Silver nanoparticles were formed in a two-step process: (1) the synthesis of silver nanoparticles seeds by reduction of Ag^+^ from AgNO_3_ by adding 1 mM trisodium citrate and (2) growing Ag seeds into larger silver nanoparticles by using an additional portion of l-ascorbic acid. SERS measurements were conducted for Rhodamine 6G using a laser wavelength of 785 nm. The optimal size of silver nanoparticles was estimated, and it was in the range of 50–70 nm; furthermore, an increase in SERS signal intensity was observed with increasing Ag nanoparticle size, up to 50 nm. Above that value, the intensity of Raman enhancement reached a plateau due to limited adsorption of the analyte on larger Ag nanoparticles and reduced scattering efficiency on the SERS substrate. However, for Ag nanoparticles with a diameter smaller than 15 nm, quantum effects that hamper the accuracy of measurements and affect the enhancement factor in the SERS must be considered. 

In our research, Ag nanoparticles tend to form agglomerates of micrometric size composed of nanometric nanoparticles. Nevertheless, the detection of pyridine by measurement of high intensity of Raman signal for SERS substrates made in 0.05 M and 0.025 M AgNO_3_ solutions was successfully performed. In addition, a study by M. Pisarek et al. [76] evaluated the geometrical parameters of silver deposits in the context of interaction with an ATO matrix. In the first step, the ATO nanotubes were prepared in a glycerol/water (50:50) mixture with 0.27 M NH_4_F by using constant voltages in the range of 10–30 V [26]. After preparation, the samples were annealed at 650 °C for 3 h. Next, the silver was deposited by two sputtering methods: (1) DC magnetron sputtering and (2) silver evaporation in a low vacuum. Magnetron sputtering and evaporation in low vacuum leads to the formation of ring-like structures around the top of ATO nanotubes. Reproducible SERS substrates were obtained for both silver deposition techniques. As a main factor that affects SERS activity, the high surface area of ATO, as well as the size and distribution of silver, was highlighted.

Pyridine was chosen as the model molecule. The estimated SERS analytical enhancement factor was ~5.3 × 10^4^ and ~2.7 × 10^2^ for ATO/Ag samples with electrochemically deposited silver in 0.05M and 0.025M AgNO_3_ solution, respectively. Those are comparable with enhancement factors received in the same analyte and condition for commercially available substrate SERSitive (AEF~2.9 × 10^3^). It was proved that the specific interaction between ATO and silver nanoparticles contributes to the total enhancement factor in SERS. In our research, one of the goals was to prepare an effective SERS substrate without using expensive methods like PVD. Although measured Raman spectra for pyridine show promising results (Figure 7), the parameters, such as adsorption efficiency on the Ag surface and detection limit, will be considered for further research.

In the presented study, the ATO—with silver nanoparticles deposited on its top—has narrow edges and, in some areas, it has fixed nanoparticles inter-distance, making it a promising substrate for SERS. 

## 4. Conclusions

In the present study, cost-effective silver electrochemical deposition and colloid silver deposition on anodic titanium oxide with two morphologies were addressed to obtain efficient SERS substrates. The main conclusions can be summarized as follows:

1. Annealing of ATO in the air atmosphere for 2 h at 450 °C with a heating rate of 5 °C/min and cooling with the furnace leads to the formation of the anatase phase. In addition, the morphology of nanotubes and nanopores was preserved.
2. Silver colloids deposited on ATO tend to form agglomerates, and the factor that increases the agglomeration of nanoparticles is both the higher concentration of reducing agent (trisodium citrate) and the presence of ammonia ions.
3. Electrochemically covered ATO/Ag from a solution of 0.05 M AgNO_3_ and 0.025 M AgNO_3_ leads to the formation of ATO/Ag composite where ATO surface covered is in 57.1–62.6% and average silver clusters size is in the range of 14.8–16.9 µm. These samples were selected for future SERS measurements.
4. The obtained SERS results show that SERS analytical enhancement factor from pyridine registered on ATO/Ag samples made electrochemically in a solution of 0.05 M AgNO_3_ and 0.025 M AgNO_3_ are ~5.3 × 10^4^ and ~2.7 × 10^2^, respectively.
5. Anodic titanium oxide modified with Ag nanoparticles is an interesting potential candidate for an efficient SERS substrate. Due to the developed surface area and specific interaction between ATO and Ag nanoparticles, this composite material is promising for future generations of active SERS substrates wherein parameters such an reproducibility and adsorption efficiency are crucial factors.

## Figures and Tables

**Table 1:** Actives modes of TiO_2_ crystallographic structures. Based on data presented in reference [62,63].

Crystal Structure	Crystal System	Space Group	Active Modes	Raman Active Lattice Vibrations [cm^−1^]	Raman Bands [cm^−1^]
Anatase	Tetragonal	$D\frac{19\;\;}{4h}$	A_1g_, 2B_1g_, 3E_g_	147, 197, 397, 517, 640	144, 197, 396, 514, 635
Rutile	Tetragonal	D$\frac{14\;\;}{4h}$	A_1g_, B_1g_, B_2g_, E_g_	144, 446, 610, 827	242, 319, 446, 610, 707, 818

**Table 2:** Morphological features of nanopore and nanotube ATO morphologies after silver electrodeposition.

	Sample	Surface Coverage (%)	Average Size of Ag Nanoclusters (nm)
**Nanopore ATO morphology**	0.1 M-AgNO_3_	65.9 ± 0.3	43,640 ± 1021
	0.05 M-AgNO_3_	62.6 ± 1.1	14,790 ± 325
	0.025 M-AgNO_3_	57.1 ± 2.1	16,940 ± 222
**Nanotube ATO morphology**	0.1 M-AgNO_3_	12.7 ± 0.3	2210 ± 67
	0.05 M-AgNO_3_	8.2 ± 1.7	68 ± 6
	0.025 M-AgNO_3_	10.5 ± 0.9	71 ± 1

**Table 3:** Morphological features of nanopore and nanotube ATO morphologies after silver colloids deposition.

	Sample	Surface Coverage (%)	Average Size of Ag Nanoclusters (nm)
**ATO nanopore morphology**	0.5 wt.% trisodium citrate-AgNO_3_	13.0 ± 2.4	550 ± 32
	5 wt.% trisodium citrate-AgNO_3_	20.4 ± 2.1	810 ± 110
	0.5 wt.% trisodium citrate-[Ag(NH_3_)_2_]^+^	17.1 ± 1.8	760 ± 274
**ATO nanotube morphology**	0.5 wt.% trisodium citrate-AgNO_3_	6.3 ± 0.1	430 ± 44
	5 wt.% trisodium citrate-AgNO_3_	44.0 ± 3.5	1270 ± 147
	0.5 wt.% trisodium citrate-[Ag(NH_3_)_2_]^+^	16.2 ± 1.4	770 ± 87

## Data Availability

The data presented in this study are available on request from the corresponding author. The data are not publicly available due to the basic character of the research.
